# Influencing factors of anthracycline-induced subclinical cardiotoxicity in acute leukemia patients

**DOI:** 10.1186/s12885-023-11060-5

**Published:** 2023-10-13

**Authors:** Xi Zhou, Yue Weng, Tiantian Jiang, Wenxin Ou, Nan Zhang, Qian Dong, Xiaoqiong Tang

**Affiliations:** 1 Department of Hematopathology, The First Affiliated Hospital of Chongqing Medical University, Yuzhong, Chongqing China; 2 Department of Cardiology, The First Affiliated Hospital of Chongqing Medical University, Yuzhong, Chongqing China

**Keywords:** Acute leukemia, Anthracycline, Subclinical cardiotoxicity, Influencing factors

## Abstract

**Background:**

Current treatment of acute leukemia is based on anthracycline chemotherapy. Anthracyclines, despite improving patient survival, have serious cardiotoxicity and therefore cardiac monitoring should be a priority. The purpose of this study is to explore the possible early predictors of anthracycline-induced subclinical cardiotoxicity(AISC)in acute leukemia patients.

**Methods:**

We conducted a prospective observational study involving 51 patients with acute leukemia treated with anthracycline. Demographic data, clinical variables, echocardiography variables and biochemical variables were collected at baseline and after 3 cycles of chemotherapy. Patients were divided into the AISC and No-AISC groups according to changes of global longitudinal peak systolic strain. Regression models and receiver operating characteristic curve analysis were used to explore the relationship between the variables and AISC.

**Result:**

17 of the patients suffered subclinical cardiotoxicity after 3 cycles of anthracycline treatment. Multiple logistic regression analysis showed a significant association of DBil (OR 0.612, 95% CI 0.409–0.916, p = 0.017), TBil (OR 0.841, 95% CI 0.717–0.986, p = 0.033), PLT (OR 1.012, 95% CI 1.002–1.021, p = 0.016) and Glu (OR 1.873, 95% CI 1.009–3.475, p = 0.047) with the development of AISC. After 3 cycles of chemotherapy, there was a significant difference in PLT between the AISC and NO-AISC groups. Moreover, the dynamic changes in PLT from baseline to after 3 cycles of chemotherapy were each statistically significant in the AISC and NO-AISC groups. The combination of PLT and N-terminal pro–B-type natriuretic peptide (NT-proBNP) had the highest area under curves (AUC) for the diagnosis of AISC than PLT and NT-proBNP alone (AUC = 0.713, 95%CI: 0.56–0.87, P = 0.017).

**Conclusion:**

Total bilirubin (TBil), direct bilirubin (DBil), platelets (PLT) and blood glucose (Glu) are independent influencing factors for AISC in acute leukemia patients receiving anthracycline therapy. Bilirubin may be a protective factor and PLT may be a contributing factor for AISC. The combination of baseline PLT and baseline NT-proBNP shows satisfactory predictive ability for AISC in acute leukemia cases treated with 3 cycles of chemotherapy.

## Introduction

Acute leukemia including acute lymphoblastic leukemia (ALL) and acute myeloid leukemia (AML) presents at all ages, from the newborn to the very old [1]. Acute leukemia remains one of the most common malignancies of the hematological system. For the treatment of acute leukemia, the anthracycline-based chemotherapy is mostly used in clinical practice, which has the advantages of strong anti-tumor effect and good efficacy. However, anthracycline (ANT) has been shown to have a high number of cardiac side effects that can pose a significant risk to patients including: left ventricular dysfunction, congestive heart failure (CHF), conduction and rhythm disturbances [2]. Consequently, cardiac monitoring should be a priority in patients with acute leukemia treated with ANT chemotherapy. Echocardiographic techniques are effective tools for early detection of cardiotoxicity, especially Tissue Doppler Imaging (TDI) and myocardial strain imaging. Myocardial strain imaging can sensitively predict early myocardial dysfunction, especially with two-dimensional (2D) speckle tracking echocardiography (STE). Two-dimensional (2D) STE is able to assess the global longitudinal peak systolic strain (GLS, %), which has become the clinical diagnostic standard. International Cardio-Oncology Society (IC-OS) recommend defining a > 12% reduction in GLS relative to baseline as an indicator of subclinical cardiotoxicity [3]. Combination of certain biomarker with GLS can improve the sensitivity of detection for anthracycline-induced subclinical cardiotoxicity (AISC). The main objective of this study is to identify some biomarkers as early predictors of AISC in the acute leukemia patients receiving ANT therapy.

## Methods

### Study population

Acute leukemia patients were recruited from the First Affiliated Hospital of Chongqing Medical University. The patients were invited to join the present study when they had the routine chemotherapy based on anthracyclines in the hospital. For subject recruitment, the inclusion criteria were Chinese older than 16 years with histologically positive diagnosis of ALL or AML, chemotherapy protocol, which involved administration of anthracyclines, and left ventricular ejection fraction (LVEF) ≥ 50%. While the exclusion criteria were severe liver or kidney dysfunction, history of myocardial diseases, coronary artery disease and congestive heart failure prior to chemotherapy, collagenosis and other systemic diseases.

Patients with AML (excluding M3) were given the standard 3 + 7 induction chemotherapy comprising daunorubicin (60 mg/m^2^d) on days 1–3 plus cytarabine (Ara-c; 100–200 mg/m^2^d) on days 1–7. Consolidation chemotherapy consisted of high-dose Ara-c-based regimens (1–3 g/m^2^ every 12 h) for a 4-week cycle for 3 ~ 6 courses. Patients with ALL were given VDLP regimen induction chemotherapy comprising daunorubicin (60 mg/m^2^d) on days 1–3 puls vincristine (1.4 mg/m^2^d) on days 1, 8, 15, 22, pegaspargase (2500 IU/ m^2^d) on days 9, 19, dexamethasone (15 mg/m^2^d) on days 1–7 and the corticosteroid dose was slowly reduced for a total use of 28 days. The combination of consolidation drugs includes drugs used for induction chemotherapy, such as vincristines, anthracyclines and corticosteroids for a 4-week cycle for 3 ~ 6 courses also. According to disease remission, risk stratification, physical status and economic level, patients could choose whether to undergo HSCT after induction and consolidation chemotherapy.

The study was registered in Chinese Clinical Trial and the date of first registration was 25/12/2021 under registration number ChiCTR2100054721. This study was conducted in accordance with the Declaration of Helsinki and approved by the ethics committee of the First Affiliated Hospital of Chongqing Medical University (Approval NO. 2018-016). Informed consent was obtained from all participated patients before the commencement of the interview and ultrasound examination.

### Echocardiographic evaluation

The echocardiographs was done by a single experienced operator. Conventional two-dimensional (2D) transthoracic echocardiography (TTE) was done to access cardiac functional and structural changes, such as left ventricular diastolic dimension (LVDd), left ventricular mass index (LVMI), fractional shortening (FS), left ventricular ejection fraction (LVEF), mitral inflow E velocity, mitral e’ velocity and E/e’. 2D speckle tracking echocardiography (STE) was used to assess global longitudinal peak systolic strain (GLS). All the echocardiograph data were evaluated in the time of baseline and after 3 cycles of chemotherapy. According to International Cardio-Oncology Society (IC-OS) consensus statement [3], subclinical cardiotoxicity was defined as normal LVEF but a relative GLS decrease from baseline of ≥ 12%. The patients were divided into the AISC and No-AISC groups based on change in GLS.

### Demographic data and serologic markers

Demographic data including age, gender, body mass index (BMI), hypertension, smoking history were collected. Possible serologic markers used for the analysis of cardiac dysfunction were: high-density lipoprotein cholesterol (HDL-C), low-density lipoprotein cholesterol (LDL-C), total cholesterol (TC), total triglyceride (TG), urea nitrogen (Ure), creatinine (Cre), uric acid (UA), cardiac troponin T (cTnT), N-terminal pro–B-type natriuretic peptide (NT-proBNP), alanine aminotransferase (ALT), aspartate aminotransferase (AST), total bilirubin (TBil), direct bilirubin (DBil), albumin (ALB), prothrombin time (PT), activated partial thromboplastin time (APTT), fibrinogen (FIB), fibrinogen degradation products (FDP), D-dimer, white blood cell (WBC), red blood cell (RBC), platelets (PLT), hemoglobin (Hb) and glucose (Glu). As the same, serologic markers were collected at baseline and after 3 cycles of chemotherapy.

### Statistical analysis

The data analysis was conducted using IBM SPSS Statistics (Version 25). Normally distributed continuous data were expressed as means ± standard deviation (SD), and was checked using independent sample t-test. Non-normally distributed continuous data were expressed as median (Q1-Q3) and was checked using Wilcoxon Mann–Whitney test or Kruskal Wallis test. Also, categorical data were expressed as n (%) and checked by the Chi-square or Fisher ’s exact test. Through single binary logistic regression analysis, variables with P<0.1 were regarded as confounding factors including PLT, UA, E and e’(age and gender were additional confounding factors). Independent AISC risk factors were determined using a multiple binary logistic regression model. All variables with P ≥ 0.1 were added one at a time in the multiple model. Odds ratios (OR) and 95% confidence intervals (CI) were calculated. A p value < 0.05 was considered to be statistically significant.

## Results

### Demographic and disease-related characteristics

The demographic and disease-related characteristics of the patients are shown in Table 1. In total, 51 acute leukemia patients were included in the study, 41 patients with AML and 10 patients with ALL. The patients included 26 males and 25 females with a median age was 43.25 ± 16.29 years. After 3 cycles of chemotherapy, 17 patients developed anthracycline-induced subclinical cardiotoxicity and 34 patients did not. Median age of the AISC group was 39.35 ± 17.2 and the NO-AISC group was 45.97 ± 15.73 (p = 0.176). After 3 cycles of chemotherapy, the cumulating dosage of doxorubicin was 270 mg/m^2^. Confounding factors including age, gender and the variables that were found statistically significant (p<0.1) in the single binary logistic regression analysis, such as UA (p = 0.08), PLT (p = 0.051), E (p = 0.059) and e’ (p = 0.052) ( Table 2).

**Table 1:** Demographic, disease-related, and baseline characteristics of acute leukemia patients completed 3 cycles of chemotherapy

Characteristics		All patients	No-AISC	AISC	P-value
		N = 51	N = 34	N = 17	
Age (year)		43.25 ± 16.29	45.97 ± 15.73	39.35 ± 17.2	0.176
gender	male n(%)	26(51)	18(52.9)	8(47.1)	0.629
	female n(%)	25(49)	16(47.1)	9(52.9)	
Leukemia type	AML n(%)	41(80.4)	30(88.2)	11(64.7)	0.105
	ALL n(%)	10(19.6)	4(11.8)	6(35.3)	
Marrow blast percentage(%)		63.5(43.25,79.5)	58(27,75.5)	73(58,84)	0.151
Sepsis during		9(17.6)	6(17.6)	3(17.6)	1
chemotherapy(%)					
Grade IV		47(92.2)	32(94.1)	15(88.2)	0.854
myelosuppression(%)					
BMI (kg/m2 )		22.35(19.18,24.8)	23.4(18.88,24.8)	21(17.9,25.7)	0.868
Smoking(%)		15(29.4)	10(29.4)	5(29.4)	1
Hypertension(%)		7(13.7)	5(14.7)	2(11.8)	1
HDL-C(mmol/L)		0.74(0.6,1.12)	0.78(0.7,1.39)	0.66(0.58,0.93)	0.303
LDL-C (mmol/L)		1.7 ± 0.65	1.69 ± 0.68	1.53 ± 0.51	0.402
TC (mmol/L)		3.1 ± 0.92	3.17 ± 0.85	2.88 ± 0.74	0.25
TG (mmol/L)		1.4 ± 0.6	1.40 ± 0.63	1.54 ± 0.89	0.506
Ure(mmol/L)		5.02 ± 1.46	4.93 ± 1.52	5.71 ± 2.12	0.136
Cre(µmol/L)		69(53,80)	67(51,79.25)	69(55,80)	0.204
UA(mmol/L)		302(256,390)	298.5(232.5,375.5)	324(271,408)	0.124
cTNT(ng/L)		4(0, 7)	0(0,6)	4(0,5)	0.382
NT-proBNP (ng/L)		144.5(79.75, 364.75)	98(76.5,194)	184(66,407)	0.201
ALT(U/L)		20(14,32)	17.5(10.25,22)	23(14,50)	0.126
AST(U/L)		18(12,34)	17(12.25,26.5)	18(12,45)	0.555
TBil(µmol/L)		9.1(5.5,13)	11.55(5.78,18.35)	6.2(4.5,10.7)	0.15
DBil(µmol/L)		2.9(1.8,4.7)	3.95(2.55,6.95)	2.1(1,3.1)	0.097
ALB(g/L)		41(37.75,43.25)	41(39,46)	41(40,43)	0.35
D-Dimer(µg/L)		1.03(0.49,2.31)	0.66(0.42,1.06)	1.45(0.41,2.27)	0.208
WBC(10^9^/L)		9.05(2.86,33.23)	21,32(2.91,43.14)	4.01(2.08,23.24)	0.246
RBC(10^12^/L)		2.64 ± 1.01	2.68 ± 1.01	2.41 ± 0.96	0.371
Hemoglobin(g/L)		84 ± 30	86 ± 30	75 ± 27	0.216
PLT(10^9^/L)		43(21,130)	40(23,95)	75(19,195)	0.184
PT(s)		12.85 ± 1.5	12.96 ± 1.65	12.86 ± 1.08	0.828
APTT(s)		28.2(25.9,33.3)	27.5(25.9,34.1)	29.3(25.8,33.2)	0.936
FIB(g/L)		3.1 ± 1.25	3.44 ± 1.39	3.97 ± 5.75	0.608
FDP(µg/mL)		2.8(1.4,6.2)	2.7(1.3,6.2)	4.7(1.4,16.8)	0.441
E(cm/s)		80.2 ± 17.2	77.4 ± 21.3.	89.8 ± 19.7	0.051
e’ (cm/s)		8.5 ± 2.3	8.4 ± 2.2	9.9 ± 2.6	**0.045**
E/e’		10 ± 2.9	9.5 ± 2.7	9.4 ± 2.8	0.855
LVDd(mm)		48 ± 4	47 ± 4	48 ± 5	0.417
LVMI(g/m^2^)		99 ± 21	96 ± 19	97 ± 24	0.829
FS(%)		36 ± 3	36 ± 3	36 ± 3	0.687
LVEF(%)		65 ± 4	66 ± 3	65 ± 4	0.614
Glu(mmol/L)		5.6(5.3,6.2)	5.6(5.3,6.2)	5.4(5.0,6.5)	0.852
LDH(U/L)		535(242,989)	464(178.5,1136.5)	535(392.5,926)	0.529

AISC: anthracycline-induced subclinical cardiotoxicity, AML: acute myeloid leukemia, ALL: acute lymphoblastic leukemia, BMI:body mass index, HDL-C:high-density lipoprotein cholesterol, LDL-C:low-density lipoprotein cholesterol, TC:total cholesterol, TG:total triglyceride, Ure:urea nitrogen, Cre: creatinine, UA:uric acid, cTnT:cardiac troponin T, NT-proBNP:N-terminal pro–B-type natriuretic peptide, ALT:alanine aminotransferase, AST:aspartate aminotransferase, TBil:total bilirubin, DBil:direct bilirubin, ALB:albumin, WBC:white blood cell, RBC:red blood cell, PLT:platelets, PT:prothrombin time, APTT:activated partial thromboplastin time, FIB:fibrinogen, FDP:fibrinogen degradation products, E:mitral inflow E velocity, e’:mitral e’ velocity, LVDd:left ventricular diastolic dimension, LVMI:left ventricular mass index, FS:fractional shortening, LVEF:left ventricular ejection fraction, Glu:glucose, LDH: lactate dehydrogenase

Values are mean ± SD, median (Q1-Q3) or n (%). Significant result (P < 0.05)

**Table 2:** Single binary logistic regression with anthracycline-induced subclinical cardiotoxicity as dependent variable

Characteristics	OR (95%CI)	P-value
Age, per 1 year	0.975(0.939, 1.012)	0.175
Gender,yes versus no	0.79(0.246, 2.537)	0.692
UA,per 1 mmol/L	1.004(0.999, 1.009)	**0.08**
PLT,per 1*10^9^/L	1.008(1, 1.017)	**0.051**
E,per 1 cm/s	1.028(0.999, 1.059)	**0.059**
e’, per 1 cm/s	1.306(0.998, 1.71)	**0.052**

OR (95% CI), odds ratio (95% confidence interval); UA:uric acid; PLT:platelets; E:mitral inflow E velocity, e’:mitral e’ velocity; DBil:direct bilirubin

Table 2 omits other variables with p ≥ 0.1

Variable entered in the multiple binary logistic regression analysis (P < 0.1)

### Independent risk factors for AISC

**Fig. 1:**
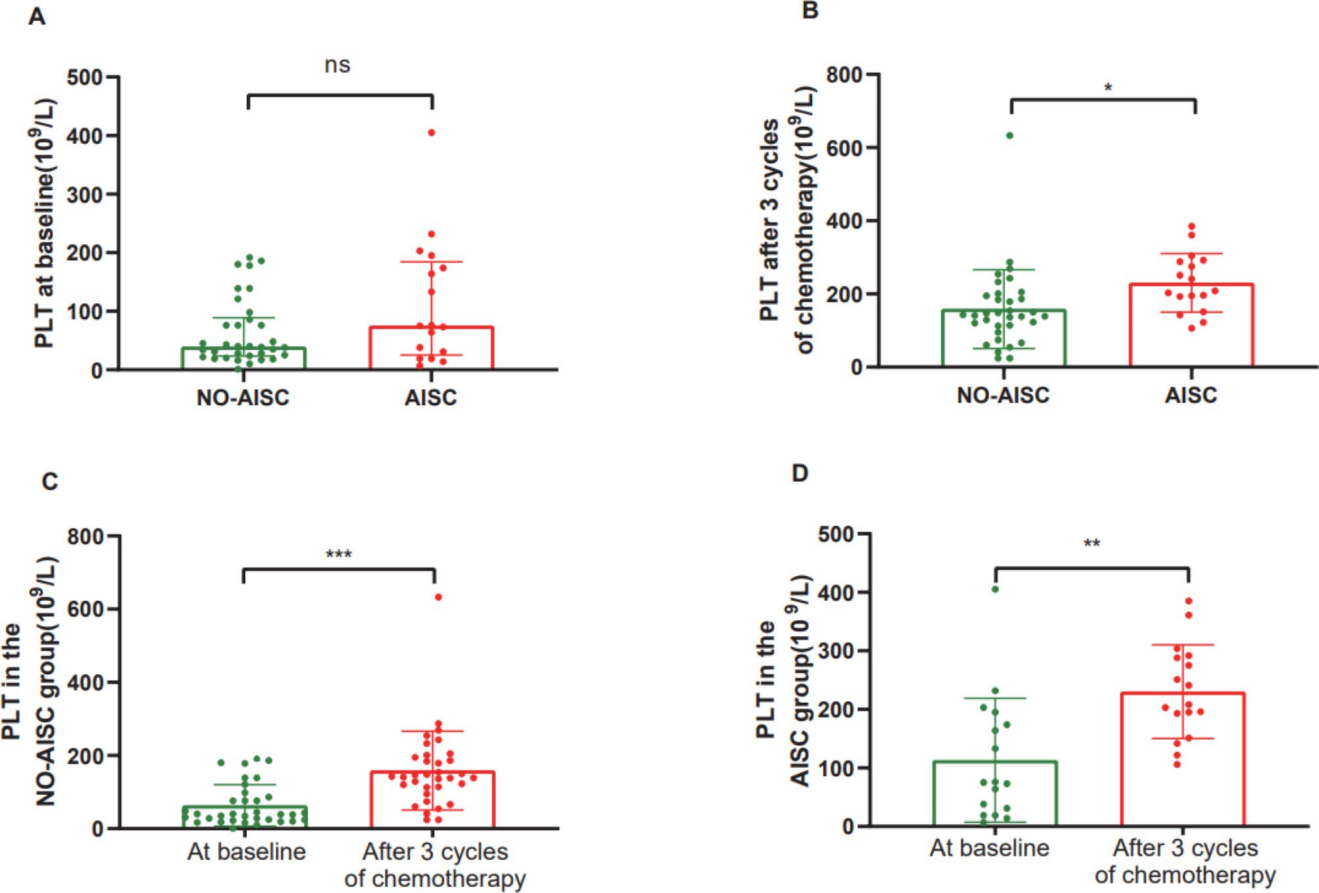
**A. B.**: Distribution and differences of PLT in acute leukemia patients in groups NO-AISC and AISC at baseline and after 3 cycles of chemotherapy, respectively. **C. D.**: The differences of dynamic change in PLT from baseline to after 3 cycles of chemotherapy in acute leukemia patients in group NO-AISC and group AISC, respectively. (AISC: anthracycline induced subclinical cardiotoxicity; PLT: platelets; ns: p>0.05; *: p<0.05; **:p<0.01; ***: p<0.001)

Incorporating confounding factors (age, gender, UA, PLT, E and e’) into the equation to create a multiple logistic regression model. All variables with P ≥ 0.1 in the single binary logistic regression analysis were added one at a time in the multiple model. DBil (OR 0.612, 95% CI 0.409–0.916, p = 0.017), TBil (OR 0.841, 95% CI 0.717–0.986, p = 0.033), PLT (OR 1.012, 95% CI 1.002–1.021, p = 0.016) and Glu (OR 1.873, 95% CI 1.009–3.475, p = 0.047) were significantly associated with AISC (multiple logistic regression, Table 3). At baseline, the difference in PLT between the AISC and NO-AISC groups was not significant (Fig. 1.A). However, after 3 cycles of chemotherapy, there was a significant difference in PLT between the AISC and NO-AISC groups (Fig. 1.B). In addition, the dynamic changes in PLT from baseline to after 3 cycles of chemotherapy were each statistically significant in the AISC and NO-AISC groups (Fig. 1.C.D).

**Table 3:** Multiple logistic regression analysis on AISC in acute leukemia patients completed 3 cycles of chemotherapy

Characteristics	OR (95%CI)(Adjusted)	P-value
Age, per 1 year	0.985(0.923, 1.051)	0.985
Gender, yes versus no	0.784(0.181, 3.408)	0.746
UA,per 1 mmol/L	1.005(0.999, 1.012)	0.102
PLT,per 1*10^9^/L	1.012(1.002, 1.021)	**0.016**
E,per 1 cm/s	1.014(0.972, 3.408)	0.516
e’, per 1 cm/s	1.195(0.691, 2.067)	0.524
DBil,per 1µmol/L	0.612(0.409, 0.916)	**0.017**
TBil,per 1µmol/L	0.841(0.717, 0.986)	**0.033**
Glu,per 1mmol/L	1.873(1.009, 3.475)	**0.047**

AISC:anthracycline-induced subclinical cardiotoxicity; OR (95% CI), odds ratio (95% confidence interval); UA:uric acid; PLT:platelets; E:mitral inflow E velocity; e’:mitral e’ velocity; DBil:direct bilirubin; TBil:total bilirubin; Glu:glucose

Significant result (P < 0.05)

### ROC curve for the diagnosis of AISC

**Fig. 2:**
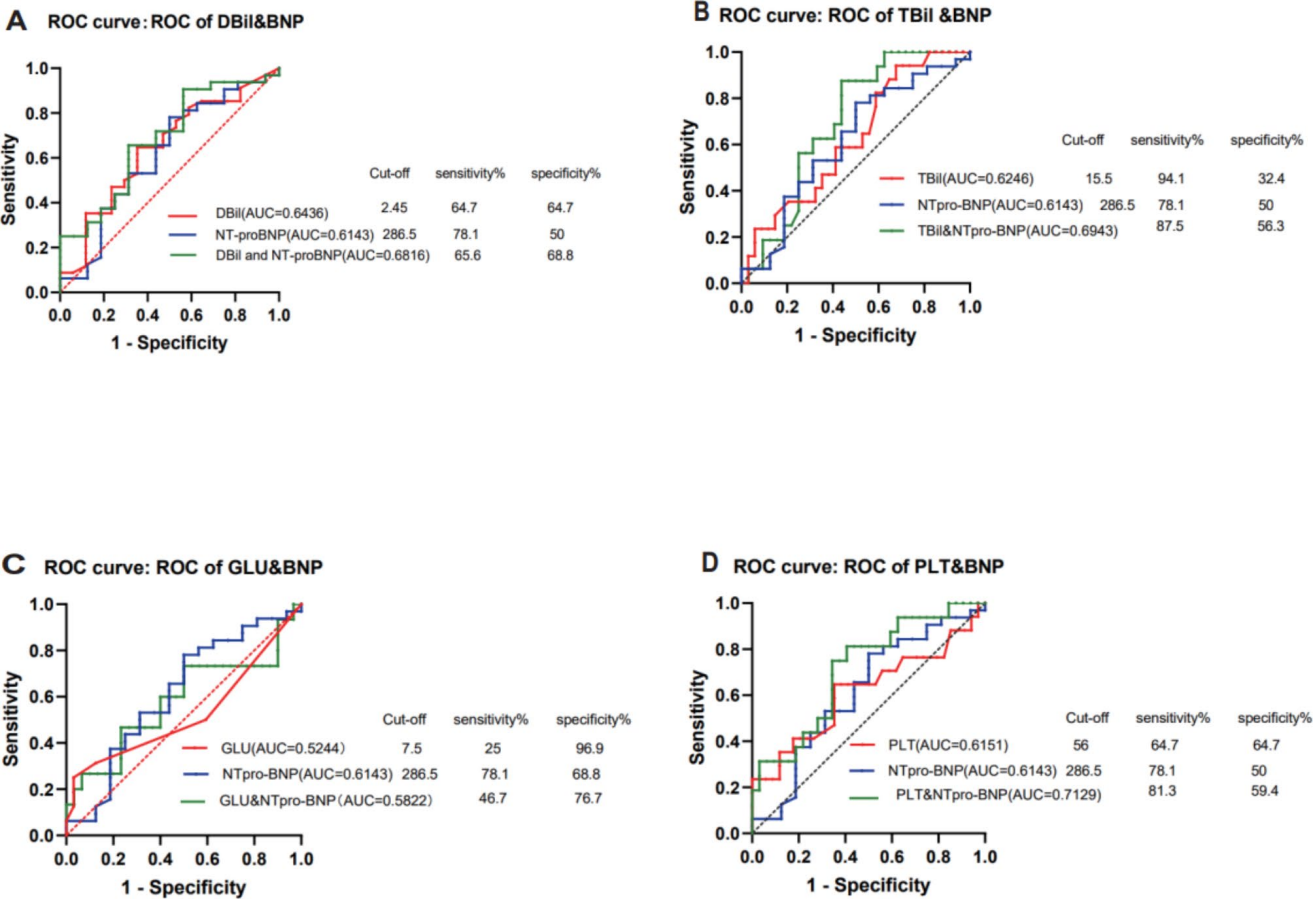
ROC curve analysis of the predictive values of DBil, TBil, Glu, PLT, NT-proBNP and their combination. (AUC: area under curves; DBil:direct bilirubin; TBil:total bilirubin; Glu:glucose; PLT: platelets; NT-proBNP:N-terminal pro–B-type natriuretic peptide)

Since DBil, TBil, PLT and Glu were significantly associated with the AISC, ROC curve analysis was conducted to determine the cutoff values. Results showed that the cutoff values of DBil was 2.45µmol/L (P = 0.097, AUC = 0.644, sensitivity = 0.647 and specificity = 0.647), TBil 15.5µmol/L (P = 0.15, AUC = 0.625, sensitivity = 0.941 and specificity = 0.324), Glu 7.5mmol/L (P = 0.85, AUC = 0.524, sensitivity = 0.25 and specificity = 0.969), PLT 56*109/L (P = 0.18, AUC = 0.615, sensitivity = 0.647 and specificity = 0.647) for detecting AISC. Previous study has shown that the NT-proBNP elevations preceded development of congestive heart failure after induction chemotherapy, concluding that NT-proBNP seems to be a promising early marker and predictor of anthracycline-induced cardiotoxicity [4]. Thus, we also used ROC curve analysis to evaluated the diagnostic validity of NT-proBNP as a cardiotoxicity biomarker. Results showed that the cutoff value of NT-proBNP was 286.5ng/L (P = 0.2, AUC = 0.614, sensitivity = 0.782 and specificity = 0.5) for detecting AISC. The AUC of both PLT and NT-proBNP were not statistically significant independently, with a p>0.05. However, their combined use provided a higher prognostic value (P = 0.017, AUC = 0.713, sensitivity = 0.813 and specificity = 0.594).(Fig. 2).

## Discussion

We have found that DBil, TBil, PLT and Glu were independent influencing factors of anthracycline-induced subclinical cardiotoxicity (AISC) in the acute leukemia patients. Bilirubin may be a protective factor for the development of AISC. Conversely, platelets may be a contributing factor to the development of AISC. The dynamic changes in platelets could better predict AISC in the acute leukemia patients. In addition, PLT and NT-proBNP combined use provided an elevated prognostic value.

Cardiac side effects associated with anthracycline treatment may seriously compromise the prognosis and well-being of patients with acute leukemia. There are currently two types of cardiotoxicity that develop secondary to chemotherapy. Type I is equivalent to trastuzumab-induced cardiotoxicity, which is characterized by a transient deterioration of myocardial function without causing cell death. This is not a dose-dependent process and is reversible in most cases, so that once myocardial function is restored, the drug can be readministered [5]. Type II is due to direct oxidative stress-induced myocardial cell death, which seems to be specific to anthracyclines. In myocardial biopsies, this is an irreversible, dose-dependent and readily identifiable process [6] .

Although anthracyclines have been studied for many years, the pathogenesis of their cardiotoxicity and the exact mechanisms leading to their cardiomyopathy have not been fully elucidated. Since the initial discovery of anthracyclines, the principal mechanism responsible of their cardiotoxic potential has been attributed to the excessive reactive oxygen species (ROS) production [7–11]. ANT induces the production of ROS through enzymatic action, redox reactions and the formation of iron complexes. The activation of the oxidative stress system and the accumulation of ROS lead to microsomal lipid peroxidation, ultrastructural changes in cardiomyocytes and mitochondrial damage, ultimately causing irreversible damage to cardiomyocytes [12].

Bilirubin is the end product of heme catabolism in mammals and high bilirubin levels are often considered to be a sign of liver damage [13]. Interestingly, recent studies suggest that bilirubin may have some cytoprotective effects [14]. Bilirubin has strong antioxidant properties and can prevent oxidative damage caused by a variety of oxidant-related stimuli [15]. Schwertner and Vitek’s study found that patients with Gilbert’s syndrome(individuals with higher bilirubin levels) had a lower incidence of heart disease than the general population [16]. Low total bilirubin level has been demonstrated to be a risk factor for cardiovascular disease [17]. In addition, studies suggest that bilirubin may help fight multidrug resistance in tumors [18]. Thus, Siqi Zhang et al. have speculated that bilirubin may have a cardioprotective effect in doxorubicin-induced cardiotoxicity. Besides, in their study, they found some possible mechanisms for the protective effect of bilirubin on doxorubicin -induced cardiotoxicity as follows: (1) bilirubin alleviates JNK-Cx43 gap junctions’ dysfunction; (2) bilirubin activates AMPK-SOCS3 axis to inhibit Cx43 phosphorylation and protect H9C2 cells against doxorubicin toxic injury; (3) Axl is involved in bilirubin-induced SOCS3 expression [19]. There is now a growing body of experimental evidence that moderately increased serum bilirubin concentration might serve as a strong chain-breaking antioxidant in biological systems, being helpful for plasma, cellular, tissue protection, and thus helping to guard against the development and progression of cardiovascular disease and other diseases related to enhanced oxidative stress [20].

Platelets are anucleate fragments of megakaryocytes which serve as the primary cellular mediator of hemostasis and monitor vascular endothelial cell injury [21–23]. Platelets can also produce reactive oxygen species(ROS), among the initial studies on platelets and oxygen free radicals is that of Marcus et al. [24] that confirmed superoxide anion production by platelets with the ferricytochrome c and nitroblue tetrazolium assays. Recent studies have suggested that activated platelets act as an potentially and additional major source of hydroxyl radicals, superoxide anion, and hydrogen peroxide, because of the biomass of platelets compared to other blood cells [25–29]. The main source of platelet ROS production is nicotinamide adenine dinucleotide phosphate (NADPH) produced in the pentose cycle, while ROS production as a by-product of mitochondrial respiration plays a secondary role [30]. However, when using anthracycline-based chemotherapy, the observations of Zhicheng Wang et al. further confirmed that doxorubicin (DOX) can induce mitochondrial ROS production in platelets [31]. The exact mechanism of how DOX leads to elevated platelet mitochondrial ROS levels is unknown. On the one hand, DOX may increase the production of mitochondrial ROS [32]. On the other hand, DOX may cause decline in mitochondrial antioxidant capacity. Platelets mitochondria produce more ROS in response to DOX [31], upregulation of ROS is strongly associated with anthracycline-induced cardiotoxicity [7–11]. Because of the malignancy itself, platelets in patients with acute leukemia are often below normal level at baseline. The cardiac benefits of anti-platelet drugs such as aspirin need to be weighed against the risk of bleeding due to thrombocytopenia.

Our study suggests that PLT may be a contributing factor to the development of subclinical cardiotoxicity in acute leukemia patients treated with anthracycline chemotherapy, which may be associated with greater platelet production of ROS. Although platelets were elevated in both NO-AISC group and AISC group after 3 cycles of chemotherapy, and the dynamic changes in platelets were both statistically significant, it was not possible to consider the dynamic change in platelets as more meaningful in predicting the AISC. Once the acute leukemia patients response to the chemotherapy (entering complete or partial remission), the platelet count would definitely return to a normal or near-normal condition and be definitely higher than that during the time of diagnosis.

Diabetes is widely recognized as an independent risk factor for cardiovascular disease. In fact, hyperglycemia can facilitate the accumulation of ROS through different metabolic pathways: (1) enhanced polyol activity, causing sorbitol and fructose accumulation; (2) increased formation of advanced glycation end products (AGEs); (3) activation of protein kinase C (PKC) [33] and (4) increased hexosamine pathway flux [34]. Hyperglycemia leads to the accumulation of ROS through various pathways, ultimately promoting cellular damage. This may be a mechanism by which blood glucose acts as a contributor to AISC.

The above studies support the conclusion of our study, which may be the mechanism of the clinical phenomenon we have discovered. In the next step, we will continue to enroll more patients and investigate possible mechanisms.

### Limitation

The study population analyzed included only 51 patients. This limitation is even more relevant in certain subgroups, such as the number of patients with AISC [17]. Moreover, our study was conducted in a single center. Due to the possible heterogeneous distribution of prognostic variables, these limitations may bias our findings. Special attention should be focused on large prospective studies in the future that clarifying whether total bilirubin (TBil), direct bilirubin (DBil), platelets (PLT), and glucose (Glu) determination can be useful in the monitoring of subclinical cardiotoxicity risk in acute leukemia patients undergoing anthracycline therapy.

## Conclusion

Evaluating the association of anthracycline-induced subclinical cardiotoxicity (AISC) during acute leukemia treatment with different factors, we have found that baseline total bilirubin (TBil), direct bilirubin (DBil), platelets (PLT) and glucose (Glu) are independent influencing factors for AISC. Bilirubin may be a protective factor while PLT may be a contributing factor for AISC. Also, we have found that the combination of baseline PLT and baseline NT-proBNP shows satisfactory predictive ability for AISC in acute leukemia patients receiving 3 cycles of chemotherapy.

## Data Availability

The data that support the findings of this study are available from the Chinese Clinical Trial Registry (http://www.chictr.org.cn/showproj.aspx?proj=145082) but restrictions apply to the availability of these data, which were used under license for the current study, and so are not publicly available. Data are however available from the corresponding author upon reasonable request.

## Abbreviations

ANT: anthracycline
GLS: global longitudinal peak systolic strain
AUC: area under curves
AISC: anthracycline-induced subclinical cardiotoxicity
ALL: lymphoblastic leukemia
AML: acute myeloid leukemia
CHF: congestive heart failure
TDI: Tissue Doppler Imaging
2D: two-dimensional
IC-OS: International Cardio-Oncology Society
TTE: transthoracic echocardiography
STE: speckle tracking echocardiography
BMI: body mass index
HDL-C: high-density lipoprotein cholesterol
LDL-C: low-density lipoprotein cholesterol
TC: total cholesterol
TG: total triglyceride
Ure: urea nitrogen
Cre: creatinine
UA: uric acid
cTnT: cardiac troponin T
NT-proBNP: N-terminal pro–B-type natriuretic peptide
ALT: alanine aminotransferase
AST: aspartate aminotransferase
TBil: total bilirubin
DBil: direct bilirubin
ALB: albumin
WBC: white blood cell
RBC: red blood cell
PLT: platelets
PT: prothrombin time
APTT: activated partial thromboplastin time
FIB: fibrinogen
FDP: fibrinogen degradation products
E: mitral inflow E velocity
e’: mitral e’ velocity
LVDd: left ventricular diastolic dimension
LVMI: left ventricular mass index
FS: fractional shortening
LVEF: left ventricular ejection fraction
Glu: glucose
OR: odds ratio
CI: confidence interval
ROS: reactive oxygen species
DOX: doxorubicin
NADPH: nicotinamide adenine dinucleotide phosphate
